# Prognostic factors associated with improvement in patients with an episode of non-specific low back pain without radicular syndrome: a prospective observational exploratory study

**DOI:** 10.1186/s12998-025-00580-5

**Published:** 2025-05-21

**Authors:** Gaetan Barbier, Martin Descarreaux, François Cottin, Arnaud Lardon

**Affiliations:** 1 CIAMS, Université Paris-Saclay, Orsay Cedex, 91405 France; 2 CIAMS, Université d’Orléans, Orléans, 45067 France; 3 Institut Franco-Européen de Chiropraxie, Ivry-sur-Seine, 94200 France; 4 Department of Human Kinetics, Université du Québec à Trois-Rivières, Trois-Rivières, Québec Canada; 5 Université Paris-Saclay, Inria, CIAMS, Gif-sur-Yvette, 91190 France

**Keywords:** Manual therapy, Chiropractic, Low back pain, Prognostic factors

## Abstract

**Background:**

Low back pain is a leading cause of disability worldwide, with most cases classified as non-specific(NSLBP). While manual therapy appears effective for treating NSLBP, further research is needed to identify candidate baseline factors associated with improvement to help tailor personalized treatment strategies. This prospective observational exploratory study, therefore, aims to identify candidate prognostic factors collected at baseline that are associated with short-term improvement in people with NSLBP.

**Methods:**

This study was conducted in chiropractic clinics across France between March 1, 2022, and February 28, 2023. Adults with a new episode of NSLBP were included. Baseline data, including individual, clinical, and therapist-related candidate factors, were collected before and during the initial consultation. Participants were considered improved if they: (i) reported “all better” or “better” on perceived global change, (ii) achieved a 20-point improvement on both Visual Analog Scales (VAS for intensity and unpleasantness) or scored 0 on reassessment, and (iii) showed a 30% improvement on the Oswestry Disability Index (ODI) at 7 days and 4 weeks post-consultation. Missing data were handled using multiple imputation with chained equations (MICE). Logistic regression analyses (univariate and multivariable with spline terms when superior fit was demonstrated) identified candidate prognostic factors associated with clinical improvement.

**Results:**

Out of 1,394 patients contacted, 241 met the inclusion criteria, and 207 completed at least one follow-up assessment. After imputation and multivariable analysis, duration of episode (spline 1: 0.94[0.89-1.00]), Number of painful sites (0.75[0.62–0.92]), negative treatment expectations (0.48 [0.25–0.94]), disability score (spline 1: 0.94[0.89-1.00], spline 2: 0.77[0.62–0.96]), and pain intensity (1.05 [1.02–1.07]) were associated with improvement at 7 days. At 4 weeks, disability score (spline 1: 1.24[1.07–1.45], spline 2: 0.77[0.63–0.95]), pain intensity (1.02 [1.00–1.04]), episode duration (spline 1: 0.95[0.91-1.00]), new patient (0.50 [0.28–0.91]), and clinican’s prognosis (3.89 [1.49–10.10]) were associated with improvement.

**Conclusion:**

Less-studied factors, such as negative treatment expectations, clinician’s prognosis, number of therapists, and perceived stiffness, highlighted significant associations with improvement in this exploratory phase. These findings suggest that incorporating these factors may be used when updating existing models.

**Supplementary Information:**

The online version contains supplementary material available at 10.1186/s12998-025-00580-5.

## Introduction

Low back pain represented the leading cause of years lived with disability for individuals of all ages and was one of the top ten causes of disability-adjusted life-year in 2019 [1]. Most cases of low back pain are classified as non-specific (NSLBP) [2, 3]. Several contributors influence an individual’s pain experience, and the disability associated with NSLBP, including biopsychosocial factors, genetic factors, comorbidities, and pain-processing mechanisms [2].

High-quality clinical practice guidelines recommend a multimodal treatment approach for NSLBP, incorporating reassurance, advice, education, and manual therapy combined with other active modalities, as well as psychological interventions for subgroups with elevated psychosocial risks, mood disturbances, or persistent pain [4, 5]. Regarding manual therapy specifically, a systematic review of recent clinical practice guidelines for managing low back pain indicates that spinal manipulation, mobilization, and massage are treatments modalities recommended for both acute and subacute NSLBP [6]. These recommendations are supported by evidence from 11 to 7 clinical practice guidelines, respectively [6].

Patients with NSLBP generally have positive attitudes and beliefs about manual therapy, viewing it as an effective and safe treatment option with manageable levels of soreness following treatment [7]. Although numerous studies have investigated the efficacy of manual therapy for NSLBP, the factors associated with clinical improvement following these interventions remain poorly understood [8]. Over 200 prognostic models have been published to predict clinical outcomes in individuals with low back pain receiving conservative treatments, including physiotherapy, exercise, manual therapy, and medication. However, these models lack sufficient evidence to be reliably used in clinical practice [9]. It seems necessary to improve prediction models for conservative treatments of NSLBP by addressing common methodological flaws, such as selection of predictors [9, 10]. The process of predictor selection is a fundamental component of the first step, exploratory prognostic studies, in the PROGRESS framework for prognosis research, which provides a structured approach to studying prognostic factors [11, 12]. It seeks to identify factors (i.e. psychological, behavioral, or biological) that can predict clinical outcomes in the absence of specific treatment. When these prognostic factors also exert causal or mechanistically relevant effects, they become strong candidates for predicting differential responses to therapy, thus contributing to the emerging paradigm of stratified medicine [13]. Initial exploratory studies, which rely on biological reasoning, hypothesized causal pathways or hypothesis-free (“biology agnostic”), are essential to identify relevant prognostic factors [12]. Such studies do not focus on one (or a few) specific prognostic factors, but rather investigate many factors and their association with health outcomes [12].

As the body of evidence grows, subsequent confirmatory work becomes essential to replicate and validate these NSLBP candidate prognostic factors, ideally using appropriate study designs. This systematic progression, in accordance with the PROGRESS framework, from initial exploration of candidate factors to rigorous evaluation, ensures that only robust prognostic factors with meaningful clinical implications can be implemented in clinical practice [11, 12].

This prospective observational exploratory study aims to identify if candidate prognostic factors collected at baseline (i.e., before or during the first visit) are associated with improvement at (i) 7 days and (ii) 4 weeks in people with an episode of non-specific low back pain without radicular syndrome.

## Methods

### Study design and setting

In this prospective observational exploratory study, we gathered candidate prognostic baseline factors (i.e. factors collected before and during the first visit) from patients presenting a new episode of NSLBP and attending either one of two chiropractic institution outpatient clinics (CC) or one of the nine affiliated chiropractic offices (CO) in France between March 1, 2022, and February 28, 2023. Ethical approval for this study was obtained from a French Committee of Protection of Persons (21.01053.000012. Cat 3 HPS - dated 05-OCTOBER-2021) Electronic and oral consent to participate was obtained from each participant before data collection, and all procedures in this study followed the principles of the Declaration of Helsinki. This article follows the recommendations of the REMARK checklist [14].

### Participants

#### Eligibility criteria

Consecutive patients with a new episode of NSLBP aged 18 years or older were enrolled. A new episode of NSLBP was defined as having pain or an increase of pain present for more than 24 h and preceded by a month without pain or stable pain [15]. 

The exclusion criteria were the following: Specific causes of low back pain (e.g. vertebral fractures, inflammatory disorders, malignancy, infections, intra-abdominal causes) [16], radicular syndrome(s) (e.g. radicular pain, radiculopathy, spinal stenosis) [16], pregnancy or breastfeeding, age < 18 years, inability to provide consent, or inability to speak French. Individuals who visited a manual therapist for low back pain in the past three months were excluded. An episode of care for low back pain is defined as a consultation or a series of consultations for low back pain preceded and followed by at least three months without consultation for low back pain [15]. 

#### Procedures

All patients who were scheduled for an appointment at the CC or CO for pain were contacted the day before their appointment by phone by chiropractor to ensure that the eligibility criteria were met (pre-enrollment). Patients were informed about the study’s aim and details during this call, and oral consent was sought. If the patient was eligible and agreed to participate, the day before the first consultation, a questionnaire was sent to potential participants by e-mail (baseline questionnaire), and electronic consent was obtained. The diagnosis of NSLBP was made by chiropractors or supervised chiropractic students. During the consultation, eligibility criteria were subsequently verified by each chiropractor or supervised chiropractic student receiving the potential participant. Eligible and consenting participants were assessed by email at baseline, after 7 days and after 4 weeks [17–19]. In cases where the questionnaire was incompletely filled out or not completed, participants received another mail 24 h later and an SMS 48 h later. Four variables were used to measure the participants’ improvement: perceived global change was assessed at 7 days and 4 weeks. The Oswestry Disability Index and the two Visual Analog Scale for pain (VAS) were completed at baseline, 7 days, and 4 weeks.

Data collection for candidate prognostic factors was done through self-reports via the baseline questionnaire and by chiropractors during the first visit. Descriptions of the chiropractors and supervised chiropractic students involved in recruitment and data collection are given in the “Treatment” section. To maximize response rates, in cases where questionnaires were partially completed, participants were contacted by phone on the day of the baseline visit. All candidate prognostic factors, and clinical outcome measures were collected similarly at all the sites, using an electronic Case Report Form (eCRF) Data Capture (Castor EDC, Netherlands, Amsterdam).

Participants who did not respond to any follow-up questionnaires were considered “baseline only”, whereas those who responded to at least one follow-up questionnaire, either at 7 days, at 4 weeks, or both, were considered “analyzed”.

### Treatment

The treatment consisted of a pragmatic chiropractic intervention including manual therapy techniques such as manipulation, mobilization, and soft-tissue therapy, along with oral education and advice. Treatments were performed face-to-face and individually, either within two chiropractic institutions outpatient clinics or in private practices. At the institution’s outpatient clinics, supervised chiropractic students conducted consultations under the supervision of licensed chiropractors with over three years of clinical experience. All students conducting consultations at these clinics were invited to participate. In private practices, treatments were carried out by 11 chiropractors with 4 to 29 years of experience who all graduated from the French Institute of Chiropractic (IFEC). The 11 chiropractors were contacted and invited to contribute by a member of the research team (GB). Each chiropractor or supervised chiropractic student participating in this study received training in data collection to ensure consistency. This pragmatic approach was tailored to each consultation (duration approximately 30 min), with chiropractors or supervised chiropractic students determining treatment plans based on individual patient needs. Adherence to treatment, education and advice were not assessed.

### Variables

#### Clinical outcome measures

The Patient Global Impression of Change (PGIC) was assessed by one item of the global self-assessment of recovery, “How do you see the recovery from your disorder?” (Seven response options from “all better” to “getting much worse”) [20, 21].

NSLBP intensity was assessed using a visual analog scale (VAS), “average intensity of pain felt today” (0–100), and NSLBP unpleasantness was assessed using a visual analog scale, *“the average unpleasantness of pain”* (0–100) [22, 23].

Disability was assessed using the Oswestry Disability Index (ODI), translated and validated into French [24, 25]. The ODI assesses how back pain affects the patient’s ability to perform daily activities. The minimum score is 0, and the maximum score is 50.

#### Improvement criteria

**Fig. 1:**
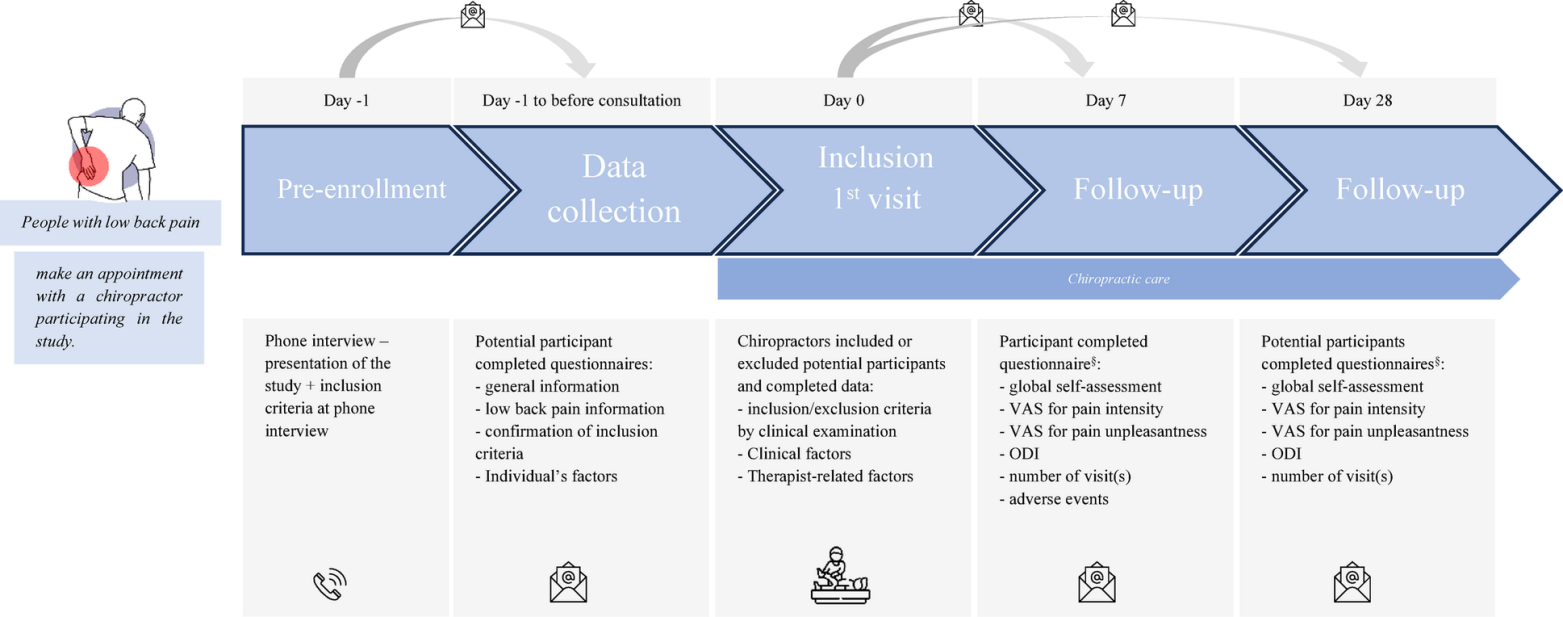
Overview of the study timeline, inclusion criteria, procedures, treatment, collected candidate prognostic factors and outcomes. *Episode of low back pain was defined as a period of work absence due to low back pain, preceded and followed by a period of at least 1 day at work| ^§^in cases where the questionnaire was incompletely filled out or not completed, participants have received another mail 24 h after and SMS 48 h after.| VAS: Visual Analog Scale| ODI: Oswestry Disability Index.| y: yes| n: no| +: painful| -: painless.

A participant was classified as improved only if they meet all three criteria: (1) they answered “all better” or “better” on the PGIC [26] (2) they improved 20 points on both VAS scales [27] or rated 0 on reassessment, and (3) they achieved a 30% improvement in disability [27]. This classification is based on Burns et al. 2018 [28], who used the classification in a study investigating factors associated with clinical improvement in individuals with a primary a complaint of low back pain. Participants not meeting these criteria were considered non-improved. The measurement times for each clinical outcome measure are shown in Fig. 1.

#### Adverse events

Adverse effects were collected on 7 days using measurement instruments to evaluate adverse events following spinal manipulation therapy [29]. A general question regarding potential adverse effects (“Did you have any problems/side effects as a result within a week of your treatment (by your chiropractor)?“) was asked to all participants. For those who reported adverse effects, a series of specific questions followed to further explore and characterize their experiences. The questionnaire used is available in Supplementary Table S1.

#### Candidate prognostic baseline factors

The candidate prognostic baseline factors were selected based on the Delphi study focusing on predictors of instantaneous relief from spinal manipulation for NSLBP [30], as well as through consultation with researchers and chiropractors participating in this study. All candidate prognostic factors are presented in Table 1. The measurement timepoints for each candidate prognostic factor are shown in Fig. 1.

**Table 1:** Candidate prognostic factors collected in baseline questionnaire or by chiropractor or supervised chiropractic student during the first visit

Prognostic factor group	Candidate prognostic factors	Type of variable	Definition	Question (measure)	Data provider
Individual’s factors	Age	Continuous	Age calculation based on date of birth	Date of birth (Years)	Participant
	Body mass index	Continuous	The BMI is calculated using the following formula: BMI = weight (kg) / (height (m)^2^)	Weight (kg) Height (m) Body mass index (BMI) categories were defined as follows: underweight (< 18.5 kg/m²), normal weight (18.5–24.9 kg/m²), overweight (25.0–29.9 kg/m²), and obesity (≥ 30.0 kg/m²).	
	Chronic disease*	Categorical	The patient has a chronic disease(s), also known as noncommunicable disease, diagnosed by a medical doctor	Do you have one or more (chronic) disease(s) for which a diagnosis has been made? (Yes/No)	
	Gender	Categorical	Gender of patient	Gender (Women, Men, other)	
	Level of education	Categorical	level of education was investigated with the highest level of diploma completed	What is the highest level of education you have completed? (: no primary school diploma or certificate, middle school, “Certificat d’Aptitude Professionnel (CAP), Brevet d’études professionnelles (BEP) or equivalent” (Career and Technical Education (CTE), ex: baker, plumber), high school, short higher education diploma (baccalauréat (bac) + 2), and long higher education diploma (higher than bac + 2).)	
	Long-term treatment	Categorical	Patient on long-term treatment	Are you taking long-term treatment for a medical condition? (Yes/No)	
	Number of painful sites	Continuous	Number of painful sites in the last 12 months	Section 1 Nordic questionnaire (0–15)	
	Widespread pain	Categorical	The patient has widespread pain	Section 1 Nordic questionnaire (Yes/No). Participants are classified as yes if it is satisfied by pain in four or five body regions in the presence of at least seven painful sites (WP2019), evaluated with Sect. 1 Nordic questionnaire) [48]	
Clinical factors	Age of first LBP	Continuous	Age of patient’s first back pain episode	Indicate the age of your first lower back pain. (Years)	Participant
	Positive treatment expectations	Continuous	Score of positive expectation about treatment	the Stanford Expectations of Treatment Scale[36]– Item 1,3,5. (1–7)	
	Negative treatment expectations	Continuous	Score of negative expectation about treatment	the Stanford Expectations of Treatment Scale[36]– Item 2,4,6.^£^ (1–7)	
	Number of therapist(s)	Continuous	Number of therapists consulted by the patient for low back pain	Indicate the number of therapists consulted for your back pain. Do not include the chiropractor you will be consulting in the next day. (Number)	
	Disability	Continuous	The Oswestry Disability Index (ODI) is a widely used, condition-specific outcome measure designed to assess the degree of disability related to low back pain.	Oswestry disability Index	
	Pain intensity	Continuous	Pain intensity experienced by the patient	indicate the pain intensity you feel in your lower back– VAS (0-100)	
	Pain unpleasantness	Continuous	Pain unpleasantness experienced by the patient	indicate the pain unpleasantness you feel in your lower back– VAS (0-100)	
	Previous episode(s) of LBP	Categorical	The patient has had previous episodes of low back pain	Have you ever had a painful episode in your lower back? (Yes/No)	
	Start Back Screening Tools	Continuous	risk for persistent disabling back pain	Start Back Screening Tools (0–9)	
	Stiffness	Continuous	Lumbar stiffness experienced by the patient.	indicate the stiffness you feel in your lower back– VAS (0-100)	
	Use of medication	Categorical	The patient is on medication for low back pain.	Are you currently taking medication to manage your low back pain? (Yes/No)	
	Episode duration	Continuous	Duration of the current episode of back pain.	Duration of the current episode of back pain. (Day: 1–90)	Chiropractor or supervised chiropractic student
	Feeling of patient’s improvement	Categorical	Feeling of patient’s improvement expressed spontaneously after treatment	Did the patient spontaneously express improvement during the post-treatment consultation? The term “spontaneous” means that you did not ask any questions about it (Yes/No)	
	Hip pain FADDIR/FABER	Categorical	Pain experienced by the patient during the FABER and/or FADIR test.	At least one of the two tests, FABER or FADIR, induces pain in the patient. (Yes/No)	
Therapist-related factors	New patient	Categorical	The patient’s status within the clinic.	1st visit in the office (Yes/No)	chiropractor or supervised chiropractic student
	Clinician’s prognosis	Categorical	Prognosis perceived by the clinicians	Based on the patient’s history, clinical examination, and feelings, the patient will improve rapidly? (strongly disagree, moderately disagree, slightly disagree, neither agree nor disagree, slightly agree, moderately agree, or strongly agree). “Moderately agree” and “strongly agree” were classified as indicating a “good” prognosis, while “strongly disagree,” “moderately disagree,” “slightly disagree,” “neither agree nor disagree,” and “slightly agree” were classified as indicating a “bad” prognosis.	

* If treatment for a chronic disease(s) was indicated in the long-term treatment question, but the participant had ticked “no” to chronic disease(s). Chronic disease(s) has been added a posteriori in this item.| ^£^ A seven-point Likert-type response scale was chosen for each question, with the following anchors: (1) “strongly disagree,” (2) “moderately disagree,” (3) “slightly disagree,” (4) “neither agree nor disagree,” (5) “slightly agree,” (6) “moderately agree,” or (7) “strongly agree”. An average of items 1, 3, and 5 yields a positive expectancy score. An average of 2, 4, and 6 yields a negative expectancy score [36]

##### Individual’s factors

Study participants completed a standardized self-report questionnaire about age, Body Mass Index (BMI) was calculated from height and weight, chronic disease, gender, level of education was investigated with the highest level of diploma completed, long-term treatment, number of painful sites and widespread pain (WSD).

##### Clinical factors

Study participants completed a standardized self-report questionnaire about current and previous episodes of low-back pain (age of first low back pain, number of therapist(s) consulted for low back pain, disability, pain intensity, pain unpleasantness, previous episode of low back pain, Start Back Screening Tools (SBST), stiffness experienced by the patient, use of medication for low back pain) and expectations about treatment [31].

During consultations, chiropractors or supervised chiropractic students collected the following candidate prognostic factors: episode duration (number of days of the episode of low back pain for which the patient consulted), physical examination of hips (FABER-FADDIR test) [32], feeling of patient’s improvement expressed spontaneously after treatment (*Did the patient spontaneously express improvement during the post-treatment consultation? The term “spontaneous” means that you did not ask any questions about it).*

##### Therapist-related factors

During the consultation, the chiropractors or supervised chiropractic students collected the following candidate prognostic factors: new patient (1st visit in the office), prognosis perceived by chiropractors or supervised chiropractic students (chiropractors or supervised chiropractic students were asked to answer “strongly disagree, moderately disagree, slightly disagree, neither agree nor disagree, slightly agree, moderately agree, or strongly agree” to the following question: Based on the patient’s history, clinical examination, and feelings, the patient will improve rapidly).

### Statistical methods

Baseline characteristics of participants were summarized using means and standard deviations (SD) for continuous variables and frequencies with percentages for categorical variables.

To handle missing data, multiple imputations using chained equations (MICE) were performed. We imputed ten datasets using 20 iterations. Predictive Mean Matching (PMM) was used for continuous variables, and logistic regression was applied for categorical variables. The primary analysis was conducted on imputed datasets. As a sensitivity analysis, we also performed all multivariable analysis using a complete-cases approach. Logistic regression analyses were performed to explore associations between candidate prognostic factors at baseline and improvement. Two analysis were constructed: (1) univariate logistic regressions, (2) multivariable logistic regression analysis that included candidate predictors adjusted by pain intensity, disability, and WSD [33]. To further explore the potential non-linear relationship between all candidate prognostic factors and improvement, restricted cubic spline (RCS) with 3 knots (10, 50, 90 percentiles) analysis were applied. Analyses with and without RCS were compared using likelihood ratio tests, and RCS were retained in the logistic regression analysis when they significantly improved regression fit. Multicollinearity among predictors was assessed by variance inflation factors (VIF), and variables showing significant multicollinearity (VIF > 5) were excluded from multivariable analysis to avoid instability in coefficient estimation [34]. The strength of associations was reported using odds ratios (OR) and 95% confidence intervals (CI).

All statistical analyses were conducted with Stata 18.0 (StataCorp LLC). Statistical significance was defined as *p* < 0.05.

## Results

### Participants

**Fig. 2:**
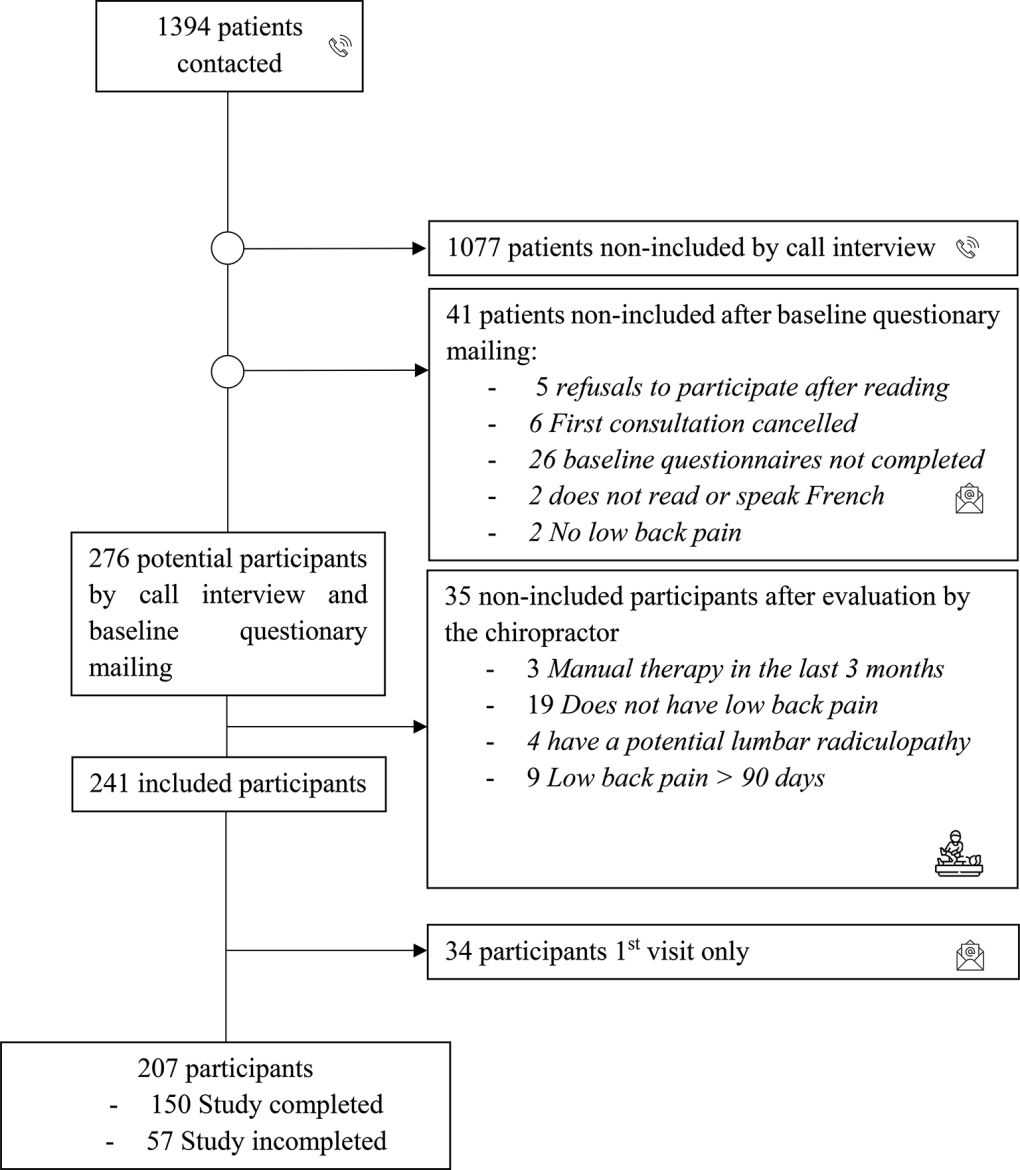
Flow diagram illustrating the participant selection process for the study

Out of 1394 patients who scheduled a chiropractic appointment, 317 were considered potential participants after the call interview, 276 remained eligible after completing the baseline questionnaire, and finally, 241 reported NSLBP, met all inclusion criteria, and were included in the study following an assessment by the chiropractors or supervised chiropractic students. Details are presented in Fig. 2– A flow diagram illustrating the participant selection process for the study.

Among the 241 participants included, 34 (14%) did not respond to any follow-up questionnaires. A total of 194 participants (80%) completed the 7-day follow-up questionnaire, and 166 (69%) completed the 4-week follow-up questionnaire.

### Baseline characteristics

The participants (*n* = 241) were, on average, 44.2 (± 15.7) years old, mostly men (*n* = 156, 65%). Details of the baseline characteristics of the included sample are shown in Tables 2 and 3.

**Table 2:** Descriptive data for continuous candidate prognostic factors

	Obs	Mean	SD	Min	Max
Age (years)	241	44.2	15.7	18	76
BMI (kg/m^2)^	241	25.8	4.7	17	57
Age of first LBP (years)	213	30.5	13.3	11	66
Episode duration (days)	239	23.0	24.4	1	90
VAS (/100)					
Intensity	241	49.1	20.1	9	100
Unpleasantness	241	57.4	21.7	3	100
Stiffness	241	52.2	25.7	0	100
ODI (/50)	238	11.0	7.0	1	43
SBST (/9)	238	3.1	2.1	0	8
Treatment expectation + (/7)	236	5.2	1.3	1	7
Treatment expectation– (/7)	236	2.6	1.8	1	7
Number of painful sites	238	2.6	1.8	0	8
Number of therapist(s)	241	2.5	2.8	0	15

Obs: Observation| Std. Dev.: Standard Deviation| Min: Minimum| Max: Maximum|

VAS: Visual Analog Scale| ODI: Oswestry Disability Index| SBST: Start Back Screening Tools

**Table 3:** Descriptive data for categorical candidate prognostic factors

	*n*
Gender	
Women	85
Men	156
Other	0
New patient	
No	109
Yes	131
Level of education	
No primary school diploma or certificate	11
Middle school	5
CAP, BEP, or equivalent*	33
High school	59
Short higher education diploma (associate degree)	37
Long higher education diploma (higher than associate degree)	97
Associated pathology	
No	199
Yes	42
Long-term treatment	
No	199
Yes	42
Previous episode of low back pain	
No	28
Yes	213
Use of medication for low back pain	
No	153
Yes	88
Hip pain with FADIR/FABER	
No	144
Yes	70
Widespread pain	
No	226
Yes	15
Feeling of patient’s improvement	
No	105
Yes	138
Prognosis perceived by the therapist	
Totally agree	106
Moderately agree	96
Some agree	29
Neutral	5
Some disagree	2
Moderately disagree	1
Totally disagree	1
“Bad”	38
“Good”	203

n: Number| * Career and Technical Education (CTE)

### Outcomes characteristics at follow-up

#### 7 days follow-up

**Fig. 3:**
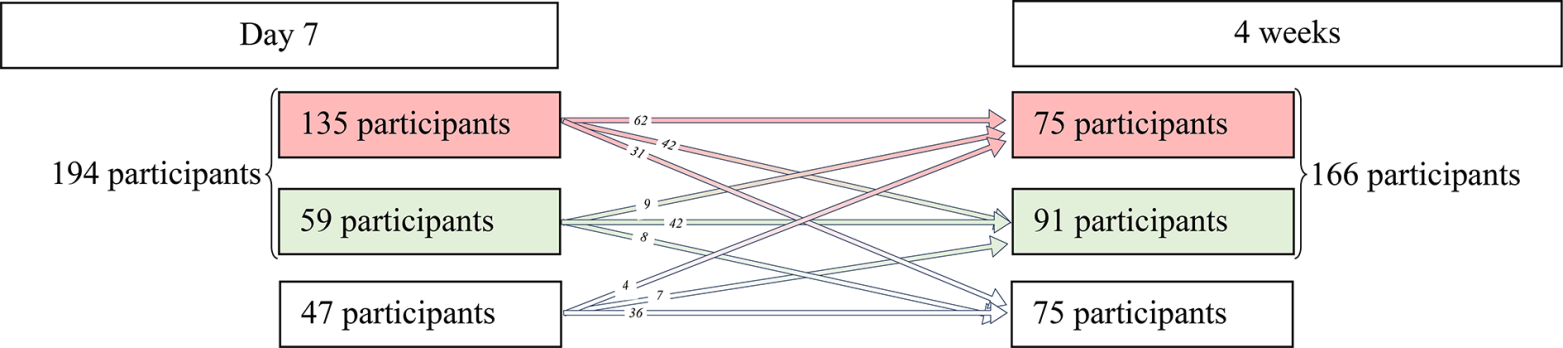
Number of participants non-improved (red), improved (green), and lost to follow-up (white) at each time follow-up

At 7 days, mean pain intensity and unpleasantness averaged 27.9 ± 19.5 [range 0.0–93.0] and 29.8 ± 22.8 [range 0.0–100.0], respectively. The mean ODI score was 6.1 ± 5.7 [range 0–35], and 46% (*n* = 111) considered themselves improved or completely improved at 7 days. In brief, 24% (*n* = 59) of the participants were considered as improved, 56% (*n* = 135) were considered as non-improved, and 20% (*n* = 47) were lost to follow-up. Two participants answered all the questions except the ODI. They could not be considered as improved or non-improved and they were categorized as lost to follow-up. Details of the numbers of participant considered as improved, non-improved, and lost to follow-up are presented in Fig. 3.

##### Adverse events

At 7 days, 40 participants (17%) described adverse effects. Among the adverse events described, the majority were discomfort/pain (*n* = 29, 73%), followed by fatigue (*n* = 16, 40%) and rigidity/stiffness (*n* = 10, 25%). No severe adverse events were reported by participants during the study. When a participant reported an adverse event, they typically reported an average of 1.8 adverse events, with the number ranging from a minimum of 1 to a maximum of 8. All adverse events are available in Supplementary Table S1.

#### 4 weeks follow-up

At 4 weeks, mean pain intensity and unpleasantness averaged 19.8 ± 18.6 [range 0.0–78.0] and 21.0 ± 20.9 [range 0.0–91.0], respectively. The mean ODI score was 4.5 ± 5.1 [range 0–36]. At 4 weeks, 46% (112) of participants considered themselves improved or completely improved. At 4 weeks, 38% (*n* = 91) of the participants were considered as improved, 31% (*n* = 75) were considered as non-improved, and 31% (*n* = 75) of the participants were lost to follow-up. Details of the numbers of participants considered as improved, non-improved, and lost to follow-up are presented in Fig. 3.

### Description of treatment modalities

During the first visit, among the 241 participants, 210 (87%) received soft tissue therapy, 207 (86%) received spinal manipulations, 160 (66%) received spinal mobilizations, 178 (74%) received advice, and 80 (33%) received therapeutic education. Between inclusion and 7 days, the average number of consultations was 1.3, ranging from 1 (inclusion consultation) to 5. Between 7 days and 4 weeks, the average number of consultations was 0.76, ranging from 0 to 4. Over the entire follow-up period, the total number of consultations varied from 1 to 8, with an average of 2.1. Details are available in Supplementary Table S2.

### Associations between candidate prognostic factors and improvement

Table 4 lists the logistic regression results used to assess the associations between candidate prognostic factors and improvement at 7 and 4 weeks.

#### Candidate prognostic factors at 7 days

Among the candidate prognostic factors, number of painful sites (OR, [95% CI] = 0.82, [0.69–0.98]), stiffness (OR, [95% CI] = 1.02, [1.00–1.03]), pain intensity (OR, [95% CI] = 1.05, [1.03–1.07]) and pain unpleasantness (OR [95% CI] = 1.04, [1.02–1.07]) were associated with improvement. Episode duration (spline 1: OR, [95% CI] = 0.94, [0.89–0.98], spline 2: OR, [95% CI] = 1.14, [1.02–1.27]) and disability score (spline 1: OR, [95% CI] = 1.29, [1.10–1.51], spline 2: OR, [95% CI] = 0.76, [0.62–0.93]) showed a significant nonlinear association with improvement. After multivariable analysis, all associations remained statistically significant except for pain unpleasantness, stiffness, and episode duration (spline 2). In addition, negative treatment expectations (spline 2: OR, [95% CI] = 0.48, [0.25–0.94]) and the number of therapists (OR, [95% CI] = 0.83, [0.72–0.95]) were significantly associated with improvement only in the multivariable analysis (see Table 4). The statistically significant results remain the same after a complete–case approach.

**Table 4:** Results of the logistic regression between candidate prognostic factors and improvement at 7 days and 4 weeks

		Day 7				Week 4			
		Univariate		Multivariable		Univariate		Multivariable	
		OR(IC95%)	p	OR(IC95%)	p	OR(IC95%)	p	OR(IC95%)	p
*Individual’s factors*									
Age (years)		1.01 (1.00-1.03)	0.13	1.00 (0.98–1.02)	0.90	1.02 (0.99–1.04)	0.15	1.01 (0.98–1.03)	0.63
BMI		0.96 (0.90–1.03)	0.28	0.93 (0.85–1.01)	0.08	0.98 (0.92–1.05)	0.59	0.97 (0.90–1.05)	0.45
Chronic disease(s) (n/y)		0.66 (0.32–1.38)	0.27	0.47 (0.20–1.10)	0.08	0.91 (0.41–2.04)	0.82	0.68 (0.28–1.68)	0.40
Gender (w/m)		1.10 (0.59–2.04)	0.76	0.93 (0.46–1.86)	0.83	1.16 (0.61–2.23)	0.65	0.97 (0.47–1.97)	0.93
Level of education (6 class)		0.97 (0.77–1.23)	0.83	1.15 (0.86–1.52)	0.34	0.84 (0.66–1.07)	0.15	0.89 (0.67–1.18)	0.41
Long term treatment (n/y)		0.79 (0.36–1.75)	0.57	0.60 (0.24–1.46)	0.26	1.00 (0.44–2.26)	1.00	0.77 (0.31–1.88)	0.56
Number of painful sites (/15)^§^		**0.82 (0.69–0.98)**	**0.03**	**0.75 (0.62–0.92)**	**0.01**	1.01 (0.86–1.20)	0.88	0.98 (0.82–1.17)	0.79
Widespread pain (y/n)		0.20 (0.02–1.67)	0.14	0.16 (0.02–1.38)	0.09	0.64 (0.19–2.14)	0.46	0.46 (0.13–1.65)	0.23
*Clinical factor*									
Age of first LBP (years)		1.02 (1.00-1.04)	0.10	1.01 (0.98–1.04)	0.45	1.01 (0.99–1.04)	0.26	1.01 (0.98–1.03)	0.63
Treatment expectations +		1.16 (0.93–1.45)	0.18	1.07 (0.83–1.38)	0.58	1.22 (0.99–1.51)	0.07	1.18 (0.94–1.49)	0.16
Treatment expectations–	*Spline 1*	0.57 (0.32–1.02)	0.06	**0.48 (0.25–0.94)**	**0.03**	0.88 (0.50–1.56)	0.67	0.79 (0.42–1.46)	0.44
	*Spline 2*	2.77 (0.87–8.84)	0.09	3.76 (0.96–14.76)	0.06	0.99 (0.33–3.01)	0.99	1.21 (0.36–4.09)	0.75
Number of therapist (s)		0.90 (0.80–1.01)	0.08	**0.83 (0.72–0.95)**	**0.01**	1.05 (0.95–1.17)	0.33	1.01 (0.90–1.13)	0.88
Disability	*Spline 1*	**1.29 (1.10–1.51)**	**0.00**	**1.22 (1.02–1.45)**	**0.03**	**1.28 (1.10–1.49)**	**0.00**	**1.24 (1.07–1.45)**	**0.01**
	*Spline 2*	**0.76 (0.62–0.93)**	**0.01**	**0.77 (0.62–0.96)**	**0.02**	**0.76 (0.62–0.94)**	**0.01**	**0.77 (0.63–0.95)**	**0.02**
Pain intensity (/100)		**1.05 (1.03–1.07)**	**0.00**	**1.05 (1.02–1.07)**	**0.00**	**1.03 (1.01–1.05)**	**0.00**	**1.02 (1.00-1.04)**	**0.04**
Pain unpleasantness (/100)		**1.04 (1.02–1.07)**	**0.00**	1.03 (1.00-1.06)	0.06	**1.02 (1.01–1.04)**	**0.01**	1.01 (0.98–1.03)	0.61
Previous episode(s) of LBP (n/y)		2.39 (0.79–7.28)	0.12	2.30 (0.65–8.13)	0.20	1.78 (0.60–5.24)	0.29	1.40 (0.41–4.82)	0.59
SBST (/9)		1.08 (0.94–1.24)	0.26	0.89 (0.72–1.09)	0.25	1.18 (1.00-1.38)	0.05	1.04 (0.83–1.29)	0.76
Stiffness (/100)		**1.02 (1.00-1.03)**	**0.01**	1.00 (0.98–1.02)	0.97	1.01 (1.00-1.02)	0.06	1.00 (0.99–1.01)	0.98
Use of medication (n/y)		1.24 (0.68–2.25)	0.48	0.77 (0.39–1.53)	0.46	**2.07 (1.05–4.07)**	**0.04**	1.58 (0.74–3.38)	0.23
Episode duration (days)	*spline 1*	**0.94 (0.89–0.98)**	**0.01**	**0.94 (0.89-1.00)**	**0.05**	**0.94 (0.90–0.99)**	**0.01**	**0.95 (0.91-1.00)**	**0.05**
	*spline 2*	**1.14 (1.02–1.27)**	**0.02**	1.12 (0.98–1.27)	0.10	**1.11 (1.00-1.22)**	**0.05**	1.08 (0.97–1.21)	0.16
Feeling of patient’s improvement (n/y)		1.42 (0.78–2.60)	0.25	1.50 (0.74–3.04)	0.27	0.98 (0.54–1.77)	0.95	0.88 (0.47–1.67)	0.70
Hip pain FADIR/FABER (n/y)		0.99 (0.52–1.86)	0.97	0.72 (0.33–1.55)	0.39	1.24 (0.60–2.57)	0.56	1.06 (0.48–2.34)	0.88
*Therapist-related factors*									
New patient (n/y)		0.65 (0.36–1.18)	0.16	0.81 (0.41–1.60)	0.54	**0.45 (0.25–0.78)**	**0.01**	**0.50 (0.28–0.91)**	**0.02**
Clinician’s Prognosis (good/bad)		1.66 (0.71–3.91)	0.25	1.81 (0.66–4.97)	0.25	**3.07 (1.30–7.26)**	**0.01**	**3.89 (1.49–10.10)**	**0.01**

Statistical test: logistic regression analysis| LBP: Low Back Pain| BMI: Body Mass Index| SBST: Score total of Start Back Screening Tools| Treatment expectation +: An average of items 1, 3, and 5 of the Stanford Expectations of Treatment Scale yields the positive expectancy score (/7)| Treatment expectation -: An average of items 2, 4 and 6 of the Stanford Expectations of Treatment Scale yields the negative expectancy score (/7)| ODI: Oswestry Disability Index| SBST: Score total of Start Back Screening Tools| n/y: no/yes| w/m: Women/Men| ^§^adjusted by intensity of pain, ODI (spline 1, spline 2)| Bold: *p* < 0.05

#### Candidate prognostic factors at 4 weeks

Among the candidate prognostic factors, new patient (OR, [95% CI] = 0.45, [0.25–0.78]), prognosis perceived by the therapist (OR, [95% CI] = 3.07, [1.30–7.26]), use of medication for low back pain (OR, [95% CI] = 2.07, [1.05–4.07]), pain intensity (OR, [95% CI] = 1.03 [1.01–1.05]) and pain unpleasantness (OR, [95% CI] = 1.02, [1.01–1.04]) were significantly associated with improvement. Episode duration (spline 1: OR, [95% CI] = 0.94, [0.90–0.99], spline 2: OR, [95% CI] = 1.11, [1.00–1.22]) and disability score (spline 1: OR, [95% CI] = 1.28, [1.10–1.49], spline 2: OR, [95% CI] = 0.76, [0.62–0.94]) showed a significant nonlinear association with improvement. After multivariable analysis, all associations remained statistically significant except for episode duration (spline 2), the use of medication for low back pain and pain unpleasantness (see Table 4). The statistically significant results remain the same after a complete–case approach.

## Discussion

This study investigated candidate prognostic factors associated with improvement at 7 days and 4 weeks in 241 patients with an episode of NSLBP without radicular syndrome. Episode duration, disability and pain intensity/unpleasantness were consistently associated with improvement at both timepoints. Additionally, the number of painful sites, negative treatment expectations, number of therapists and stiffness were also associated with improvement at 7 days, whereas at 4 weeks, new patient status, and clinician’s prognosis were associated with improvement. There is a nonlinear relationship between (i) disability score at baseline, (ii) episode duration and improvement at 7 days and 4 weeks. There is also a nonlinear relationship between negative treatment expectations and improvement at 7 days. Indeed, the exploratory nature of the study suggests that these findings should be used in new confirmatory prognostic factor studies.

Among the factors associated with improvement at 7 days and 4 weeks, de Zoete et al. identified, in a systematic review, pain intensity and episode duration as a moderator of the effect of spinal manipulative therapy (SMT) versus recommended interventions for pain and function [37]. Specifically, patients with higher level of pain and less than one year of low back pain reported more favorable clinical outcomes with SMT than with recommended interventions, including pharmacological treatments (e.g., NSAIDs, analgesics) and non-pharmacological treatments (e.g., exercise). This review noted that treatment preferences and expectations were insufficiently explored due to limited, unavailable, or study-level-only data in the included studies. Nevertheless, a secondary analysis of randomized control trials found that patients with higher positive expectations of recovery reported significantly reduced pain and improved function after chiropractic care for recurrent persistent LBP [38]. These findings align with a Cochrane review that included 60 studies and over 30,000 participants, which concluded that positive recovery expectations are associated with faster return to work, pain reduction, and increased functional capacity [39].

The present study highlighted associations between the sensation of stiffness, the number of painful sites, and improvement. To our knowledge, these factors have never been explored as potential factors associated with improvement following manual therapy treatment. However, data exists on the measurement of stiffness at baseline, either by a machine or manually, and the potential association with improvement. The initial stiffness level measured by a machine or manual does not seem to be associated with improvement following manual therapy [40–43]. Only one study suggests that the initial stiffness level is associated with a change in the ODI score at 7 days [44], and another suggests that responders to SMT for low back pain are characterized by an immediate and sustainable decrease in spinal stiffness [42]. In the present study, we explored the sensation of stiffness using a VAS ranging from 0 to 100. A cross-sectional survey was conducted to understand how patients perceive stiffness compared to discomfort and pain [45]. Patients described stiffness primarily as a limitation in movement and mobility, characterized by tightness and restricted range of motion. Unlike pain, which was perceived as more intense and harmful, stiffness was seen as less severe but still impactful, leading to functional limitations [45]. This differentiation between these two variables highlights clinicians’ need to understand these nuanced patient perspectives to improve communication and treatment strategies. The use of stiffness as a prognostic factor in clinical practice remains premature, and our findings can serve as a basis for future replication and confirmation studies.

Concerning the number of painful sites, in accordance with our results, a cohort study by Rundell et al. found that is common among older adults with persistent back pain and is associated with worse outcomes in terms of disability, pain intensity, and quality of life [46]. A systematic review published in 2019, including 34,492 individuals, revealed that LBP-related disability in relation to co-occurring musculoskeletal pain highlighted the number of pain sites that increase, reduce the probability of recovery, and decrease workability [47]. In contrast, our results did not show an association between WSD and improvement and, whereas a systematic review previously identified WSD as a generic prognostic factor in musculoskeletal pain conditions. This discrepancy is likely attributable to differences in the definitions used. In our study, WSD was defined according to WP2019 criteria [48], while in the systematic review, the authors considered multisite pains as indicative of WSD. Consequently, it may be beneficial for the management of patients with NSLBP for clinicians to take the presence of multisite pain into consideration [33, 47].

Our results revealed that patients who had previously visited their chiropractor’s clinic had a higher probability of being classified as improved at 4 weeks. This suggests that patients who improved after visiting a chiropractor may be more likely to seek care again during subsequent episodes of NSLBP. Furthermore, in 2020, a Delphi study with 19 expert chiropractors and manipulative physiotherapists identified 18 clinical predictors indicating that patients may likely experience immediate pain relief from high-velocity low-amplitude (HVLA) spinal manipulation therapy [30]. According to this expert agreement, the highest-rated predictor was a history of positive response to previous spinal manipulation. Other factors related to the practitioner’s opinion were identified, such as the practitioner’s view of the patient’s overall health (excellent to good), the practitioner’s understanding of the patient’s expectations or goals, and a good therapeutic relationship between the practitioner and the patient. The perception of a good “prognosis perceived by the therapist” was significantly associated with responder status at 4 weeks in our study. Currently, we lack data to understand what guided therapists in responding to this question, but the variables described in this Delphi study are likely involved. Further studies are needed to understand what guides therapists in perceiving patient prognosis. In our study, the therapist’s perception of a good “prognosis” was significantly associated with improvement at 4 weeks. However, we currently lack data to fully understand the factors guiding this perception. It is plausible that variables highlighted in the Delphi study, such as patient-practitioner rapport and prior treatment outcomes, are relevant. Further research is necessary to elucidate the elements contributing to the therapist’s assessment of patient prognosis.

Finally, a meta-analysis published in 2024 [49] showed that specific effects of conservative low back pain treatment accounted for only 33% of the improvement in pain, 34% of the improvement in function, and 11% of the improvement in quality of life. In contrast, natural progression accounted for around 50% of improvement in these three categories. This suggests that the factors associated with improvement in our study may reflect the natural course of recovery rather than the specific effects of manual therapy, or a combination of both.

### Limitations

As with all the experiments, our study had limitations; 20% and 31% of the included participants were lost to follow-up at 7 days and 4 weeks, respectively. To limit the number of participants lost to follow-up, in the event of a no-response, a reminder e-mail was sent 24 h later, and an SMS was sent 48 h later to encourage the participant to respond. The lost to follow-up and exclusion criteria (e.g., no manual therapy visit within the past three months) could affect the generalizability of the findings. A limitation of our study arises from the potential differences observed in prognostic factors between participants who completed the entire follow-up and those who responded either to none or only to a single follow-up questionnaire. Such differences could lead to selection bias limiting the external validity and generalizability of our findings. To address this limitation, multiple imputation was employed to account for missing data, thus mitigating potential selection bias and enabling more robust and generalizable inference. The exclusion criteria (e.g., no manual therapy visit within the past three months) may introduce selection bias and limit the generalizability of our findings.

The second limitation is that an exploratory employed does not allow us to differentiate between a moderator and a prognostic factor. This study is an exploratory study, according to PROGRESS [12], and the results can be used to generate hypotheses that need to be tested to determine whether the factor is a prognostic or moderating factor. Some of the variables used in this study were not previously validated (e.g. Clinician’s prognosis and feeling of patient’s improvement) and to our knowledge, were employed for the first time in research, (e.g. Feeling of patient’s improvement). While these variables were selected based on potential clinical relevance and consensus, the lack of prior validation may impact their reliability and interpretability. Additionally, it is possible that some patients experienced an improvement but did not explicitly express it to the therapist, leading to a potential underestimation of this variable and introducing a reporting bias. Future studies should aim to validate these measures to enhance their robustness and applicability in clinical research.

Finally, a potential limitation of our study is related to the use of a composite responder-based outcome, which might underestimate clinical improvement by classifying patients who improve substantially in certain dimensions but fail to meet predefined thresholds across all dimensions as non-responders. However, this approach offers a more comprehensive and clinically meaningful assessment by integrating multiple relevant aspects of patient improvement.

One of the strengths of this study lies in its multicenter design; 11 CC/CO participated in this project, and all potentially eligible patients were contacted. The aim was to recruit subjects with a geographical distribution close to France’s general population. The clinical outcome measures used are known to be reliable and reproducible (VAS, ODI, and PGIC) individually [20–25]. However, the composite score is not validated, although similar composite scores have been used in previous studies [28]. For instance, a multicontextual model of recovery described by Burns et al. combines the ODI, Numeric Pain Rating Scale, and global rating of change, requiring participants to meet thresholds on all measures to be classified as recovered [28]. Similarly, our composite score incorporates pain intensity, disability, and perceived recovery to provide a more comprehensive evaluation of improvement. This approach was chosen to capture multiple facets of low back pain, which are known to influence clinical outcomes.

Nevertheless, the composite score is not validated even though other previous studies have used similar composite scores. It was deemed important to use a score evaluating several components of low back pain to determine the response to manual therapy. This composite score considers pain experience, disability, and perception of recovery in people with low back pain. The therapeutic modalities of each first consultation were collected to describe the care received by the participants precisely, and adverse effects were also collected at 7 days.

### Clinical and research implications

The findings of this prospective exploratory observational study provide preliminary indications regarding factors associated with improvement in patients with NSLBP. Initial exploratory evidence regarding prognostic factors must be replicated in confirmatory studies that are adequately powered, registered, and protocol-driven, ideally with a pre-specified statistical analysis plan [50], before integration into clinical practice. These confirmatory studies should also replicate findings across diverse populations and clinical contexts to validate the robustness and generalizability of the observed associations. While promising, the current findings are intended primarily to generate hypotheses and orient future research; thus, their immediate translation to clinical decision-making remains premature without rigorous validation. Our exploratory approach considered both prognostic factors common to established models and previously underexplored factors. Future research should address this by evaluating whether the prognostic factors identified herein improve upon or complement existing prognostic models, thereby clarifying their incremental value and clinical utility. In a subsequent analysis, we will examine factors significantly associated with adverse events, specifically identifying variables contributing to their occurrence.

## Conclusion

In accordance with previous findings, factors identified as predictors of improvement including disability, pain intensity/unpleasantness, episode duration, and number of painful sites were confirmed to be associated with improvement in the present study. Additionally, other less studied factors, such as negative treatment expectations, clinician’s prognosis, number of therapists, and perceived stiffness, also highlighted significant associations with improvement in this exploratory phase. These findings suggest that incorporating these factors may be used when updating existing models or implementing confirmatory studies.

## Electronic supplementary material

Below is the link to the electronic supplementary material.

Supplementary Material 1

Supplementary Material 2

## Data Availability

No datasets were generated or analysed during the current study.

## Abbreviations

BMI: Body Mass Index
CC: Chiropractic institution outpatient Clinic
CO: Chiropractic Office
NSLBP: Non-specific low back pain
ODI: Oswestry disability outcome
PGIC: Patient global impression of change
RCS: Restricted cubic splines
SBST: Start back screening tools
SMT: Spinal manipulative therapy
VAS: Visual analog scale
WSD: widespread pain
