# Supplementary material for: Prognostic factors associated with improvement in patients with an episode of non-specific low back pain without radicular syndrome: a prospective observational exploratory study
Source: Chiropr Man Therap. 2025 May 21;33:21. doi: 10.1186/s12998-025-00580-5 (PMC12096604; doi:10.1186/s12998-025-00580-5)
Supplement: Supplementary file 1 — Supplementary Material 1 [file 12998_2025_580_MOESM1_ESM.docx]

**Supplementary**

Table S1:

Adverse events questionnaire

| - Did you have any problems/side effects as a result within a week of your treatment (by your chiropractor)?   - No   - Yes | | | | | | | | | | | | | | |
| --- | --- | --- | --- | --- | --- | --- | --- | --- | --- | --- | --- | --- | --- | --- |
| - - - If yes : | | | | | | | | | | | | | | |
|  | Discomfort/  pain | Stiffness | Weakness | Tiredness/  Fatigue | Headache | Dizziness | Vision  Problems | Problems sleeping | Difficulty Talking | Nausea/  Vomiting | Tingling/  Numbness | Strain/sprain | Difficulty  walking | Other :  ……………… |
| **1) Wich side effect(s) did you have ?** *(check all that apply)* |  |  |  |  |  |  |  |  |  |  |  |  |  |  |
| For each symptom you have, please answer the questions below and select either Yes or No. | | | | | | | | | | | | | | |
| **2) Did you expect this to occur ?** | - Yes - No | - Yes - No | - Yes - No | - Yes - No | - Yes - No | - Yes - No | - Yes - No | - Yes - No | - Yes - No | - Yes - No | - Yes - No | - Yes - No | - Yes - No | - Yes - No |
| **3) Does it interfere with your usual daily activities (e.g. work, school) ?** | - Yes - No | - Yes - No | - Yes - No | - Yes - No | - Yes - No | - Yes - No | - Yes - No | - Yes - No | - Yes - No | - Yes - No | - Yes - No | - Yes - No | - Yes - No | - Yes - No |
| **4 Does it limit your ability to care for yourself (e.g. bathing, dressing, eating) ?** | - Yes - No | - Yes - No | - Yes - No | - Yes - No | - Yes - No | - Yes - No | - Yes - No | - Yes - No | - Yes - No | - Yes - No | - Yes - No | - Yes - No | - Yes - No | - Yes - No |
| **5) Did you need to see a medical doctor because of it ?** | - Yes - No | - Yes - No | - Yes - No | - Yes - No | - Yes - No | - Yes - No | - Yes - No | - Yes - No | - Yes - No | - Yes - No | - Yes - No | - Yes - No | - Yes - No | - Yes - No |
| **6) Were you admitted to hospital because of it ?** | - Yes - No | - Yes - No | - Yes - No | - Yes - No | - Yes - No | - Yes - No | - Yes - No | - Yes - No | - Yes - No | - Yes - No | - Yes - No | - Yes - No | - Yes - No | - Yes - No |
| **7) How many hours after the therapy did it start ?** | ……. h | ……. h | ……. h | ……. h | ……. h | ……. h | ……. h | ……. h | ……. h | ……. h | ……. h | ……. h | ……. h | ……. h |
| **8) For how many days did it last ?** *(Indicate 8 if it is still continue.)* | ……. d | ……. d | ……. d | ……. d | ……. d | ……. d | ……. d | ……. d | ……. d | ……. d | ……. d | ……. d | ……. d | ……. d |
| *Pohlman KA, O’Beirne M, Thiel H, Cassidy JD, Mior S, Hurwitz EL, Westaway M, Ishaque S, Yager J, Vohra S: Development and validation of providers’ and patients’ measurement instruments to evaluate adverse events after spinal manipulation therapy. European Journal of Integrative Medicine 2014, 6(4):451-466.* | | | | | | | | | | | | | | |

Prevalence of adverse events reported by participants after the first visit (day 7).

|  |  | n | % |  |  |  |
| --- | --- | --- | --- | --- | --- | --- |
| Number of participants declared adverse events | | 40 | 21% |  |  |  |
| Type of adverse events | |  |  |  |  |  |
|  | Discomfort/pain | 29 | 73% |  |  |  |
|  | Stiffness | 10 | 25% |  |  |  |
|  | Weakness | 2 | 5% |  |  |  |
|  | Tiredness/fatigue | 16 | 40% |  |  |  |
|  | Headache | 4 | 10% |  |  |  |
|  | Dizziness | 1 | 3% |  |  |  |
|  | Tingling/numbness | 1 | 3% |  |  |  |
|  | Nausea/vomiting | 1 | 3% |  |  |  |
|  | Difficulty walking | 1 | 3% |  |  |  |
|  | Strains/Sprains | 4 | 10% |  |  |  |
|  | Problems sleeping | 3 | 8% |  |  |  |
|  | Vision problems | 0 | 0% |  |  |  |
|  | Difficulty Talking | 0 | 0% |  |  |  |
|  | Others *(heat in the back)* | 1 | 3% |  |  |  |
| * When a participant reported an adverse event \| 189 participants responded to the adverse events questionnaire | | | |  |  |  |
