# Supplementary material for: Prognostic factors associated with improvement in patients with an episode of non-specific low back pain without radicular syndrome: a prospective observational exploratory study
Source: Chiropr Man Therap. 2025 May 21;33:21. doi: 10.1186/s12998-025-00580-5 (PMC12096604; doi:10.1186/s12998-025-00580-5)
Supplement: Supplementary file 2 — Supplementary Material 2 [file 12998_2025_580_MOESM2_ESM.docx]

**Supplementary**

Table S2: Intervention characteristics at the first visit

|  |  |  |
| --- | --- | --- |
|  | n | % |
| Soft tissus therapies | 210 | 87% |
| Spinal manipulations | 207 | 86% |
| Advices | 178 | 74% |
| Spinal mobilizations | 160 | 66% |
| Therapeutics education | 80 | 33% |
